# Impact of Prior Traumatic Life Events on Parental Early Stage Reactions following a Child's Cancer

**DOI:** 10.1371/journal.pone.0057556

**Published:** 2013-03-14

**Authors:** Krister K. Boman, Ylva Kjällander, Staffan Eksborg, Jeremy Becker

**Affiliations:** 1 Department of Women's and Children's Health, Childhood Cancer Research Unit, Karolinska Institutet, Stockholm, Sweden; 2 Aastrid Lindgren Children's Hospital, Stockholm, Sweden; 3 Department of Psychology, Stockholm University, Stockholm, Sweden; University of Tasmania, Australia

## Abstract

**Background:**

In pediatric oncology, effective clinic–based management of acute and long–term distress in families calls for investigation of determinants of parents' psychological response to the child's cancer. We examined the relationship between parents' prior exposure to traumatic life events (TLE) and the occurrence of posttraumatic stress symptoms (PTSS) following their child's cancer diagnosis. Factors mediating the TLE–PTSS relationship were analyzed.

**Methodology:**

The study comprised 169 parents (97 mothers, 72 fathers) of 103 cancer diagnosed children (median age: 5,9 years; range 0.1–19.7 years). Thirty five parents were of immigrant origin (20.7%). Prior TLE were collated using a standardized questionnaire, PTSS was assessed using the Impact of Events–Revised (IES–R) questionnaire covering intrusion, avoidance and hyperarousal symptoms. The predictive significance of prior TLE on PTSS was tested in adjusted regression models.

**Results:**

Mothers demonstrated more severe PTSS across all symptom dimensions. TLE were associated with significantly increased hyperarousal symptoms. Parents' gender, age and immigrant status did not significantly influence the TLE–PTSS relationship.

**Conclusions:**

Prior traumatic life–events aggravate posttraumatic hyperarousal symptoms. In clinic–based psychological care of parents of high–risk pediatric patients, attention needs to be paid to life history, and to heightened vulnerability to PTSS associated with female gender.

## Introduction

Cancer is the most common illness–related cause of death for young people aged 1 to 19 in the United States [1]. A child's cancer diagnosis results in emotional, social, and economic consequences for the whole family [2]–[4]. For parents, the child's diagnosis and illness entails a number of potential stressors. The severity and potential fatality of the illness fulfills the diagnostic criteria for posttraumatic stress disorder as defined in the Diagnostic and Statistical Manual of Mental Disorders (DSM–IV) [5]. The period immediately following diagnosis is usually the most taxing psychologically, although a majority of parents have been found to suffer from the consequences of the child's illness and treatment long after a successful cure [6]–[9]. Although psychological vulnerability in connection with a child's illness has been thoroughly studied, there has been less investigation into the potential *determinants* of the traumatic stress reactions in parents.

Over the past decades, research perspectives have changed regarding children with chronic illness and their families. From an interest in how parental reactions are influenced by illness and treatment factors, attention has increasingly been paid to a wider range of life background factors, including the way in which individual historical, social or psychological resilience factors influence parents' experience of their child's cancer [10]–[12]. One theoretical model is Wallander and Varni's [13], which is based on risk and resistance factors in children's and parents' psychological adjustment to chronic physical disorders. Both disease–related and non–related factors influence the variability in psychosocial adaptation. Adjacent studies have indicated that life events do have an influence on psychological distress symptoms, although the degree and specificity of such relationships is yet not fully understood [14]–[16].

In the literature, the term “life events” refers to both negative and positive experiences. One event perceived as negative by one person may, by another, be perceived to be positive, or neutral [17]. According to vulnerability theory, an individual who has suffered negative life events will be more vulnerable and less resilient when faced with new distressing events; each event increasing vulnerability due to prolonged neurobiological stress responses encoded into the biochemical system [18]. Studies indicate that both earlier life experiences and on–going stress can increase vulnerability in connection with a new traumatic event. [19]–[25]. The study of parental reactions to a child's cancer in relation to past life events is warranted, since individuals have been found, after repeated traumatic life events (TLE), to be at risk for developing symptoms of accumulated, chronic, or complex posttraumatic stress resulting from prolonged stress or when facing repeated trauma or illness [26]–[28]. Based on models for addressing life events in research and clinical contexts [24], [29], assessments in this study addressed events that are highly likely to be perceived as threatening.

The central focus in the study was therefore on the relationship between parents' earlier traumatic life events and the new event constituted by their child's cancer diagnosis. Apart from the number of prior TLE experienced by each parent, three further factors were investigated as potential mediators of the relationship between TLE and posttraumatic stress symptoms (PTSS), namely parent gender, immigrant origin, and parental age at diagnosis.

Gender has been found to be a potential determinant of parents' psychological reactions to the child's illness, influencing the relationship between determinant factors and distress outcomes [10], [16], [30]–[32].

It has also been proposed that parents with an immigrant background are more prone to psychological stress than non-immigrants [33], possibly due to prior life events. Immigrant parents have demonstrated higher stress levels than non–immigrants when facing their child's cancer [34].

Since the *number* of past life events was used as the primary determinant for current cancer–related stress symptoms, we considered the age of the parent at the time of the child's diagnosis as a potential mediating factor regarding TLE and PTSS. Since older parents are likely, with time, to have experienced a greater number of TLE than younger ones, we hypothesized that the experience of more TLE among older parents would have bearing on outcomes.

### Aims

The aim was to examine the relationships between previous traumatic life experiences and the severity of stress reactions in parents of children with a newly diagnosed potentially fatal illness; more specifically, the manner in which the occurrence of earlier traumatic life events (TLE) predicted severity of parental posttraumatic stress symptoms (PTSS) in parents of recently diagnosed children.

A second aim was to investigate the influence of potentially mediating factors on the TLE–PTSS relationship: parent gender, immigrant origin, and parental age at diagnosis.

Studies of caregivers' reactions to a child's cancer have often only addressed mothers, resulting in an incomplete understanding of the impact of the cancer on families. The deliberate incorporation of both mothers and fathers in this study was aimed at gaining a more complete picture of parental reactions, permitting an analysis of outcomes by gender as well.

## Methods

### Participants

Participants consisted of 169 parents (97 mothers and 72 fathers) of the families of 103 children with newly diagnosed malignancy, registered at the childhood cancer treatment center of Astrid Lindgren Children's Hospital in Stockholm. During the investigation period, parents were assessed for eligibility and consecutively included in the study following the registration of the children at the center at the time of their first cancer diagnosis. Mothers and fathers of children diagnosed with a malignancy were evaluated for eligibility. Exclusion criteria included those whose child's illness at the time of the study was known to be incurable, although *a priori* this excluded only cases of a pontine glioma diagnosis. Parents of children in palliative care, and of deceased children, were also excluded. Furthermore, those not understanding Swedish were excluded, due to the range of languages spoken by parents with immigrant backgrounds, and questionnaires being available only in Swedish. Knowledge of Swedish was considered insufficient if parents used an interpreter in communicating with the medical staff. Information concerning immigrant status was derived from a question in the questionnaire where respondents could indicate whether they were of immigrant/non-Swedish or Swedish origin. Sociodemographic characteristics of the parent group are presented in Table 1.

**Table 1:** Sociodemographic characteristics or participating parents.

	*N*	Missing^a^	Proportion	Age, years median	Age range (min-max)
Age		11			
- Mothers	97		57.4%	38.0	34.0 (21.0–55.0)
- Fathers	72		42.6%	38.0	33.0 (22.0–55.0)
Immigrant background		0			
- Non-immigrant	134		79.3%	38.0	34.0 (21.0–55.0)
- Immigrant	35		20.7%	40.0	28.0 (27.0–55.0)
Socio-economic level^b^		5			
- Level 1 (highest)	36		21.3%	38.0	25.0 (30.0–55.0)
- Level 2 (intermediate)	50		29.6%	37.0	29.0 (26.0–55.0)
- Level 3 (lowest)	78		46.2%	40.0	33.0 (21.0–54.0)
Parents in dyad^b^ where both responded	37	0	21.9%	37.0	34.0 (21.0–55.0)
Parents in dyad where one responded	132	0	78.1%	43.0	25.0 (28.0–53.0)
No of children/parent, median = 2		1	-	-	5 (1–6)

a Missing data in variable.

b Two parents of a common child.

On average, parents had been informed about their child's diagnosis within 10 weeks prior to the study. Most children were in active cancer treatment phase, while a smaller number, mostly children with central nervous system tumors, had completed treatment. Distribution of parents and children by cancer sub–diagnosis is presented in Table 2.

**Table 2:** Children and parents in relation to diagnosis, age at diagnosis, and median time from diagnosis to study.

	Children				Parents					
			Age years				Age years		Months since diagnosis^a^	
Diagnosis	*N*	%^b^	Median	Min-Max	*N*	%	Median	Min-Max	Median	Min-Max
Leukaemia	43	42	5.6	0.1–17	72	42	37.0	21–55	1.3	0.5–6.7
Lymphoma	13	13	13.5	0.6–16.8	21	12	44.0	36–55	1.3	0.7–6.3
Central nervous system	12	11	9.0	1.3–14.9	16	10	41.5	24–52	2.6	1.0–11.8
Sympathetic nervous system	6	6	1.9	0.0–9.2	12	7	33.0	27–53	4.0	1.6–8.5
Retinoblastoma	2	2	0.8	0.5–0.8	3	2	35.0	32–43	2.3	1.6–3.8
Renal tumors	10	10	3.2	0.3–16.4	16	10	36.0	26–52	1.5	0.3–11.8
Bone tumors	6	12	10.6	9.6–19.1	10	6	43.0	40–53	1.2	0.8–7.0
Soft tissue sarcoma	5	5	10.6	0.1–17.8	8	5	38.0	33–53	1.4	0.6–12.8
Germ cell tumors	1	1	1.4	–	2	1	39.0	37–41	1.83	1.8–1.8
LCH^c^	5	5	5.2	0.3–15	9	5	38.0	30–52	3.0	1.2–3.8
Total	103	100	5.9	0.0–19.1	169	100	38.0	21–55	1.6	0.3–12.8

a Time elapsed from diagnosis to assessment.

b Valid per cent.

c Langerhans Cell Histiocytosis.

### Assessments

Earlier life events of a traumatic nature were identified using a questionnaire based on the models presented by Holmes and Rahe [24] and Bruga & Cragg [29]. It consisted of 13 categories of life events selected to include only those highly likely to be threatening, such as serious illness, injury or death of family members or close friends, physical or sexual abuse, divorce, exposure to physical violence, traffic accident, serious family conflict, and being sacked from a job. Parents were asked to mark the number of events experienced, when they occurred, and whether each event was perceived as negative or neutral/non–negative. They were also asked to add any other serious experiences not exemplified in the list. However, frequently missing information about event date made manageable analysis and presentation difficult. For this reason, event dates could not be considered, and the total number of negatively perceived events constituted the individual life events score used in analyses in this study.

The 22–item Impact of Event Scale–Revised (IES–R) was used for assessing posttraumatic stress symptoms (PTSS). The IES, designed to measure stress in relation to psychological trauma, has shown good psychometric properties when evaluated in cross-validation and reliability analyses [35]–[36] and has repeatedly been used for assessing traumatic stress in parents of childhood cancer patients, e.g., [8], [37]–[39]. While instruments addressing PTSS can be used for psychiatric diagnostic classification, it was used here only because of its general suitability for investigating parental stress reactions in the traumatic situation following a child's cancer diagnosis. The scale covers three dimensions of stress: intrusion, avoidance and hyperarousal, corresponding to the B, C and D criteria of PTSD according to DSM–IV [5]. *Intrusion* covers recurrent distressing and intrusive thoughts and experiences (8 items); *avoidance* covers avoidant behaviors, such as feelings of detachment or estrangement and avoidance of certain thoughts and feelings (6 items); and *hyperarousal* covers symptoms such as insomnia, hypervigilance, oversensitivity, and concentration problems (6 items).

Along a five–point Likert–scale scored 0–4, respondents indicated the degree to which they had suffered during the past week from the symptoms described. Parents answered using their child's cancer as the reference event.

Background information about the number of children per parent (including patient and siblings) and socioeconomic status was collected as part of the questionnaire package. Socioeconomic status was determined by classifying parents according to a system based on educational and occupational criteria, developed by Statistics Sweden for use in surveys and studies in the social field [40]. Parents were grouped into three socioeconomic levels: 1 (highest), 2 (intermediate), and 3 (lowest).

### Statistical analyses

Descriptive and summary group–level outcomes were presented for life events, and for the traumatic stress symptom categories. The interrelationships between study variables were addressed in an initial, explorative Pearson correlation analysis covering TLE, PTSS categories, and the three mediating factors addressed in this study: *parent gender*, *immigrant status*, and *age of parent*. Association between background variables, socioeconomic level, number of children/parent, and TLE and PTSS were explored in Spearman rank and Pearson bivariate correlation analyses.

TLE and PTSS outcomes were compared for mothers and fathers using two–tailed t–tests, complemented by two–way ANOVA adjusted for possible dependency between data provided by two parents of the same child.

Main analyses addressed the relationship between number of previous traumatic life events and severity of current posttraumatic stress symptoms. Firstly, the relationship was addressed in univariate regression analyses. In a following step, the predictive significance of prior TLE for parental PTSS was analyzed in two regression models adjusted for the meditating covariates (parent gender, immigrant status and age). In these two models, separate analyses were carried out for mothers, fathers, and for the entire parent group. The first model was adjusted for parent gender, and the second was adjusted for parent gender, immigrant status, and parent age.

To further examine any interaction between potential modifying factors and TLE on PTSS, a univariate two–way ANOVA was used. If an interaction effect occurred (defined by an observed tendency *p*<0.10) between a modifier and TLE, the nature of the interaction was determined by further inspection of the data. The proportion of mothers and fathers among responders and non–responders was compared using the Fisher's exact test. SPSS© 18.0 for Windows (SPSS, Inc., Chicago, Illinois) was used for the statistical evaluation. *p*–values less than 0.05 were considered statistically significant. All reported *p*–values were from two–sided tests.

### Procedures

The study was part of a larger research project investigating the psychosocial consequences of illness and treatment for families following a child's cancer diagnosis. Eligible parents received a personal invitation to participate together with detailed written information about the study. Questionnaires were mailed to be completed at home, and returned in a postage–paid reply envelope. Fathers and mothers each received their own questionnaires and were instructed to complete them separately. The study was approved by the Regional Ethics Committee.

## Results

Of the 240 parents approached, 169 (70%) provided data; 97 mothers and 72 fathers of 103 children. Parents' ages ranged from 21 to 55 years, and 35 parents came from immigrant backgrounds. The proportions of mothers and fathers differed between responders (mothers 57%; fathers 43%) and non–responders (mothers 34%; fathers 66%, *p* = 0.002). Mean time passed since diagnosis was 2.4 months (median 1.6 months, Table 2).

Parent-reported information regarding their child's treatment at the time of the study was available for 167 parents. Numbers of parents according to treatment situation were the following: no cancer treatment, 2 parents (1%), standard cancer treatment, 147 parents (88%), other treatments, 6 parents (4%), and completed cancer treatment, 12 parents (7%).

Among respondents, a majority (132) were dyads where both parents of a child responded (dyad-responders, 78%, of which 50% mothers), while 37 responders were one member of a dyad where the other parent did not respond (single responders, 22%, of which 83% mothers) (Table 1). Exposure to past TLE was similar for dyad-responders and single responders, with a tendency for a greater number of past TLE among single responders (p = 0.06). PTSS were similar for single and dyad responders regarding PTSS total score and symptom sub-categories, except for Avoidance, where dyad responders scored higher (*p*<.0.05).

On average, parents had 2–3 children (including patient and siblings, mean 2.36, median 2, Table 1). Neither of the background variables (number of children, socioeconomic level of parents) was significantly correlated with number of negative TLE or any of PTSS symptom measures.

Analysis of reported types of TLE showed that the two most commonly experienced negative life events were serious illness or injury of close relative (31%), and death of close friend or family member (23%). Other events were far less common, ranging from 0.6% for sexual abuse to 7.5% for own serious illness or injury. To examine whether there were differences in mothers' and fathers' appraisal of events (experiencing an event as negative or not negative/neutral), we analyzed the two most commonly reported events: serious illness/injury of close relative, and death of close friend/family member. The outcome showed that there was no difference between mothers' and fathers' appraisals, and that these events were hardly ever experienced other than as negative.

The frequency of experienced TLE and the PTSS outcomes are presented in Table 3. Mothers showed significantly higher symptom levels than fathers for intrusion, hyperarousal, and total PTSS scores on the IES–R. However, there was no difference between mothers and fathers as regards prior TLE or avoidance PTSS.

**Table 3:** Exposure to previous traumatic life events, and traumatic stress symptoms at follow–up.

	All parents (*N* = 169)	Mothers (*N* = 97)		Fathers (*N* = 72)		Mothers/Fathers comparisons^a^	
	Mean (SD)	Mean (SD)	Correlation with TLE	Mean (SD)	Correlation with TLE	Mean difference (95% CI)	*p* a
Number of Traumatic life events	3.6 (3.4)	3.65 (3.5)	–	3.50 (3.4)	–	0.15 (−0.93 to 1.22)	0.770
Intrusion^b^	21.1 (7.2)	22.5 (7.1)	0.03	19.3 (7.0)	0.23	3.24 (1.07 to 5.42)	0.006
Avoidance^b^	13.9 (5.1)	14.2 (5.6)	−0.08	13.5 (4.3)	−0.03	0.63 (−0.90 to 2.15)	0.090
Hyperarousal^b^	13.4 (5.2)	14.5 (5.5)	0.16	12.0 (4.4)	0.22	2.54 (1.01 to 4.07)	0.003
PTSS total score	48.4 (15.0)	51.3 (15.8)	0.04	44.6 (13.0)	0.19	6.58 (1.99 to 11.18)	0.003

a *p*–values, mothers and fathers compared regarding TLE and PTSS, adjusted for effect of potential dependency within parent couples.

b PTSS symptom category.

Inter-correlations between number of TLE and PTSS (Table 4) showed, for TLE and hyperarousal symptoms, a statistically significant positive association (*r* = 0.18, *p* = 0.02 considered here as moderate), and between TLE and intrusion symptoms, a weaker statistically non-significant positive association (*r* = 0.11, *p* = 0.15), while avoidance symptoms were unrelated to TLE. Correlations further indicated a marginally significant positive relationship between TLE and parent age (*r* = 0.15, *p* = 0.06), and none between TLE and the other two potentially mediating factors, gender and immigrant status (Table 4). The associations between gender and PTSS indicated higher PTSS levels in mothers (Tables 3 and 4), and in older parents a tendency for less hyperarousal symptoms (*r* = −0.14, *p* = 0.092) and lower total PTSS scores (*r* = −0.14, *p* = 0.078, Table 4).

**Table 4:** Inter-correlations between study variables.

	Traumatic life events	Intrusion	Avoidance	Hyperarousal	Total PTSS^a^	Parent gender	Parent age
	r (*N*)	r (*N*)	r (*N*)	r (*N*)	r (*N*)	r (*N*)	r (*N*)
Intrusion	0.113 (164)						
Avoidance	−0.062 (164)	0.461** (166)					
Hyperarousal	0.181* (162)	0.773** (164)	0.538**(164)				
Total PTSS	0.095 (162)	0.907** (164)	0.749** (164)	0.899** (164)			
Parent gender	−0.021 (166)	−0.224** (166)	−0.061 (166)	−0.241** (164)	−0.217** (164)		
Parent age	0.153 (155)	−0.090 (155)	−0.125 (155)	−0.137 (153)	−0.143 (153)	−0.101 (158)	
Parent immigrant status	0.063 (166)	0.001 (166)	0.055 (166)	0.014 (164)	0.017 (164)	0.032 (169)	0.119 (158)

* Correlation is significant at the 0.05 level (2–tailed).

** Correlation is significant at the 0.01 level (2–tailed).

a PTSS = Posttraumatic stress symptoms.

### Regression

**Figure 1:**
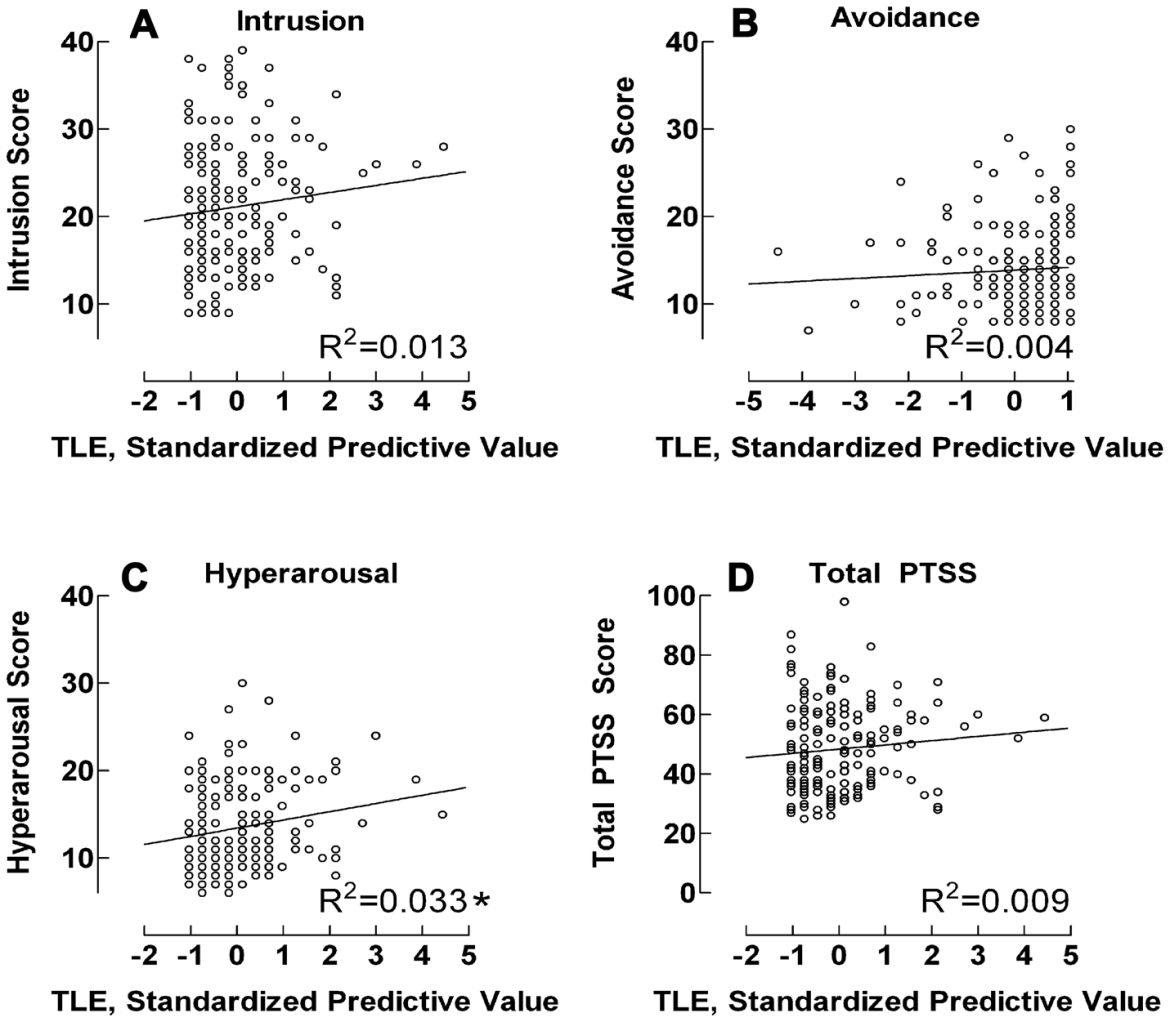
Regression–derived standardized line estimates for relationship between traumatic life events and PTSS. * = regression P<0.05. PTSS = Posttraumatic stress symptoms. Y–axis = traumatic life events; x–axis = parental cancer–related posttraumatic stress symptoms.

In the univariate regression analyses, with TLE as the predictor variable, a significant association with hyperarousal symptoms was found (B = 0.27, β = 0.18, t_161_ = 2.32, *p* = 0.021), while no association, or statistically significant one, was indicated for remaining symptom outcomes (intrusion *p* = 0.15, avoidance *p* = 0.42, total PTSS *p* = 0.23). The outcomes are illustrated in Fig. 1, showing regression plots together with corresponding *R* ^2^ determination coefficients. Together with crude scores, the standardized regression plots indicated a general although moderate pattern, where TLE tended, in varying degrees, to predict parental cancer–related traumatic stress except in the case of avoidance symptoms, the pattern being most prominent regarding hyperarousal symptoms.

In the first of two adjusted regression models (Model I, adjusted for gender in the entire group analysis), TLE predicted intrusion PTSS among fathers (B = 0.478, β = 0.234, t_68_ = 1.97, *p* = 0.053), and hyperarousal in the entire group analysis (B = 0.265, β = 0.176, t_161_ = 2.33, *p* = 0.021, Table 5)

**Table 5:** Regression outcomes for life–events as principal predictor, and posttraumatic stress symptoms outcomes.

				Model I^a^			Model II^b^		
PTS symptom category	Group	*N*	Mean score (SD)	Slope (95% CI)	*p* c	*p* d	Slope (95% CI)	*p* c	*p* d
Intrusion	Mothers	95	22.5 (7.1)	0.06 (−0.36 to 0.48)	0.780	–	0.14 (−0.30 to 0.57)	0.540	0.340
	Fathers	69	19.2 (7.0)	0.48 (−0.01 to 0.96)	0.053	–	0.27 (−0,35 to 0,88)	0.390	0.780
	Entire group	164	21.1 (7.2)	0.23 (−0.09 to 0.54)	0.15	0.005	0.15 (−0.20 to 0.50)	0.150	0.046
Avoidance	Mothers	95	14.2 (5.6)	−0.13 (−0.46 to 0.20)	0.200	–	−0.01 (0.35 to 0.33)	0.950	0.340
	Fathers	69	13.4 (4.3)	−0.40 (−0.03 to 0.27)	0.800	–	−0.23 (−0.61 to 0.15)	0.230	0.170
	Entire group	164	13.9 (5.1)	−0.09 (−0.32 to 0.14)	0.420	0.450	−0.11 (−0.37 to 0.14)	0.380	0.360
Hyperarousal	Mothers	94	14.5 (5.5)	0.25 (−0.07 to 0,57)	0.120	–	0.34 (−0.00 to 0.68)	0.052	0.150
	Fathers	68	11.9 (4.4)	0.28 (−0.03 to 0.59)	0.070	–	0.23 (−0.16 to 0.63)	0.240	0.520
	Entire group	162	13.4 (5.2)	0.27 (0.04 to 0.49)	0.021	<0.001	0.30 (0.04 to 0.55)	0.022	0.002
Total PTSS	Mothers	94	51.3 (15.8)	0.18 (−0.75 to 1.12)	0.700	–	−0.46 (−0.51 to 1.43)	0.350	0.230
	Fathers	68	44.3 (13.0)	0.70 (−0.22 to 1.61)	0.130	–	0.23 (−0.94 to 1.39)	0.700	0.930
	Entire group	162	48.4 (15.1)	0.39 (0.27 to 1.05)	0.240	0.007	−0.32 (−0.41 to 1.05)	0.390	0.025

Model I and Model II were employed separately for 3 groups: Mothers, Fathers, and Entire group.

a In Model I, the regression covering the Entire group was adjusted for parent gender.

b In Model II, the separate regressions covering Mothers and Fathers respectively, were adjusted for immigrant status and age. Regression covering the Entire group was adjusted for parent gender, immigrant status, and age of parent.

c *p*–values for the unique contribution of TLE in the model.

d Model summary *p*–value (entire model, including covariates.

Model II was adjusted for all studied mediating factors except gender in the separate analyses for mothers and fathers, and for all studied potential mediating factors in the entire group analysis. Here TLE predicted hyperarousal PTSS, although only in the group of mothers (B = 0.34, β = 0.22, t_152_ = 1.97, *p* = 0.052), and in analysis involving the entire parent group (B = 0.295, β = 0.184, t_150_ = 2.318, *p* = 0.022, Table 5).

In both Model I and Model II, model–summary statistics (from entire–group analyses with covariates entered) were significant for the intrusion, hyperarousal and the PTSS total score, apparently due to the effect of TLE combined with the effect gender.

### Mediating factors

The effect of the modifying factors of parent gender, immigrant status and age on the TLE–PTSS relationship was estimated by analyzing interaction in ANOVA. This was first done with TLE as the predictor variable and hyperarousal as the dependent, hyperarousal being the only symptom category that was clearly predicted by life events in the fully adjusted regression, Model II. No interaction between gender and TLE was demonstrated, implying that fathers and mothers did not differ regarding the predictive significance of TLE on the occurrence of hyperarousal symptoms. There were no significant interaction effects concerning parents' immigrant/non-immigrant status, nor parent age; neither was there a significant interaction between these modifying factors and TLE in subsequent analyses, with intrusion and total PTSS as dependent outcomes (avoidance was not analyzed due to the lack of relatedness to TLE).

## Discussion

This study investigated the relationship between prior traumatic life events (TLE) and the occurrence and severity of PTSS in parents of children with a recent cancer diagnosis. It was hypothesized that in this parent population, more frequent exposure to earlier TLE would be reflected by stronger PTSS in response to the child's illness. Results supported our hypothesis by verifying an association between prior TLE and current cancer–related stress regarding intrusion and hyperarousal symptoms in both the entire group, and for mothers and fathers separately.

Mothers presented stronger intrusion and hyperarousal symptoms, and higher total PTSS than fathers, thus exhibiting higher stress than fathers in response to the child's cancer. The assumption that older parents could be more vulnerable to stress due to more frequent exposures to TLE was not supported by the findings. Neither did we find support for the hypothesis that immigrant parents are at greater risk for cancer–related stress compared with non–immigrants.

### Life events

Prior life events were related to parental reactions by predicting primarily hyperarousal symptoms, rather than the whole array of PTSS. Facing the diagnosis and treatment of a child's life–threatening illness can give rise to stress reactions of the kind characteristic of posttraumatic stress disorder, PTSD [5]. In this study, however, the IES-R questionnaire was used as a useful tool to investigate parents' reactions to the sudden and traumatic change in their life situation following their child's cancer diagnosis, rather than to evaluate PTSD indications. Also, the cancer situation differs in crucial ways from many other kinds of TLE. For example, the “event”, although defined by a starting point with the cancer diagnosis, has no definite conclusion marking its end. The extended period between diagnosis and the point in time when success of lengthy treatment and subsequent long–lasting clinical follow–up can be evaluated is characterized by on–going stress and intermittent, emotionally taxing events [3], [8], [34]. This fact may explain why vulnerability following a child's cancer may be more likely to result in hyperarousal symptoms such as hypervigilance, sleep–disturbances, irritability and difficulty concentrating, than in symptoms of intrusion (such as disturbing recollections of single traumatic events) or avoidance. Hyperresponsiveness, particularly, has been found to be associated with posttraumatic symptoms [41], and hyperarousal has been found to be often associated with generic anxiety and depression in comparable populations [37]. Although the child's diagnosis occurred at least 1.5 months previously for most parents in this study, assessment of avoidance symptoms and partly also intrusion, if measured from a posttraumatic viewpoint, needs to take into account the fact that, for a majority of parents at an early stage following the child's diagnosis, treatment is still ongoing. This often means frequent treatment sessions and hospital visits, a situation that entails a kind of repeated intrusion as regards the reality of the ongoing illness, while, at the same time, both psychological and practical avoidance is hardly possible.

### Gender differences

The fact that mothers, on average, experience greater stress following the child's illness supports earlier findings [34], [42]–[43]. Differences in gender roles may contribute to this, as mothers have been found to be generally more responsible for childcare in the illness situation, and to bond more strongly with their children than fathers do [2]–[3].

The predictive significance of TLE for illness–related stress was, however, similar for both mothers and fathers, although in the fully adjusted regression model (Model II) we found that TLE predicted hyperarousal symptoms at the *p* = 0.05 level among mothers only. Our data do not offer an explanation for this finding. A plausible assumption is that this in some way corresponds to heightened stress symptoms in mothers found both in this study and in many earlier ones [32], [44]–[45].

### Immigrant status

In this study, thirty–five parents came from an immigrant background. Previous studies have found that immigrant status is a potential mediator of parents' responses to childhood cancer [34]. However, our hypothesis that this would also be reflected in a confirmed relationship between TLE and PTSS remained unsupported by our data. An explanation for this may be found in the fact that the number of TLE and severity of PTSS did not differentiate immigrant parents from parents of Swedish origin. The fact that only immigrants fluent in Swedish could participate have most likely influenced findings. The language criterion probably favored those who have lived longer in Sweden, are better integrated in society, have a better established social network and support, are better acquainted with social and healthcare services, or come from a neighboring country where the language closely resembles Swedish. Also, the parent group contained a relatively small subset of foreign–born participants—too few, perhaps, to permit a reliable analysis, or reliably reflect possible trends in the data. Knowledge of TLE and childhood cancer-related PTSS in the entire immigrant population is an important focus for forthcoming research. Assessment instruments in a variety of languages would make it possible to address a more representative immigrant study group in these future studies.

### Parent age

At the time of the study, the ages of the parents ranged from 21 to 55 years. We thought that older parents, having possibly experienced a greater number of life events, including traumatic ones, might therefore be more vulnerable to stress due to the child's illness than younger parents. However, our findings did not verify any association between parental age and PTSS levels. The lack of an association between parent age and PTSS may be explained by the fact that poorer psychological adaption in general in this population has occasionally been found to be associated with younger parental age [46]. If valid, this phenomenon could have counteracted the increased TLE-induced distress vulnerability in older parents suggested in our hypothesis. Also, other factors potentially related to older age of parents may counteract the hypothesis that vulnerability increases with number of TLE, e.g. better support networks, greater flexibility in adjusting one's routines to accommodate the on-treatment period, more life experience and consequently better coping skills.

### Limitations

A limitation of the study is the fact that the data are based on parental self–assessment alone, without support from other sources, thus risking the general bias related to the question of reliability of self–reported data. Other issues concern the limited size of the study sample—due to our wanting to approach parents close in time to the child's diagnosis—and the diagnostic heterogeneity of the children, since cancer–type has been suggested as a mediator of parental distress, playing a role in the severity of the burden of the cancer “event” [47]. In general, the findings indicate that in a larger and more varied group, the vulnerability to late PTSS reactions due to TLE might have been more explicit. The fact that the study includes only parents who understand Swedish is another limitation, with a possible bearing on the conclusions about the significance of immigranthood in the study. Our finding that the immigrant group did not show particular indications of complex or cumulative trauma may be due to the limiting criteria for inclusion of immigrant parents, such as the requirement of understanding Swedish, which tended to exclude, for example, immigrants from less well-educated or well-resourced backgrounds, and recent refugee arrivals from conflict areas. Finally, covariates that may also mediate the effect of TLE on PTSS outcomes, such as factors related to family constellation, mental or physical health, and available support from social networks, was not examined in this study.

### Clinical implications

Our findings lend support to the hypothesis that previous TLE may increase parents' vulnerability for developing PTSS symptoms when facing a child's potentially fatal illness, indicating that attention should be paid to parents' history of exposures to traumatic life events when implementing appropriate psychosocial care. In terms of parent care, awareness of life history facilitates the identification of those who may require particular attention due to heightened psychological risk. Screening for TLE and subsequent vulnerability to reactions of heightened or cumulative stress in parents could be carried out in conjunction with early hospital contacts as part of the standard collection of information about family, social network, and parents' preparedness for coping with the changed life situation. The psychosocial inquiry could include a standardized inventory of the type used in this study, which in a short format focused on major life experiences only, i.e. events that are likely to be of great traumatic significance. Because of its convenience and brevity screening with the used questionnaire could be easily incorporated into clinical routine. During treatment and follow up, special psychosocial attention could then be paid to parents identified as being specifically vulnerable to augmented reactions due to the effect of repeated trauma or cumulative stress. Awareness of the fact that, for some parents, particular difficulties in coping with the child's cancer due to prior TLE can be especially helpful in providing support in situations known to be particularly distressing or threatening, such as painful procedures, serious complications and drawbacks during treatment [48].

Gender–related explanations apply not only to difference in vulnerability for PTSS in parents. The discovery that mothers and fathers may differ regarding how life history contributes to the way distress develops soon after facing the child's diagnosis has additional implications for psychosocial care.

There was no significant influence of immigrant status on the relationship between TLE and PTSS. However, due to the selective criteria for inclusion of immigrant participants, the group of non-Swedish parents in this study was hardly representative of the entire immigrant population. Therefore, as long as our knowledge is incomplete regarding this issue, immigrants with a suspected history of traumatic experience should be addressed in clinical screenings for vulnerability to adverse psychological effects of cumulative stress.

## Conclusions

In this study of parents of newly diagnosed children with cancer, the gender–related disposition towards vulnerability regarding PTSS when facing a child's illness shows that mothers are at higher risk compared to fathers. The experience of prior traumatic life events appears to increase vulnerability to hyperarousal stress symptoms in parents who, in the early phase following diagnosis, react to their child's illness. The findings point to the importance of discerning life history and taking gender–related vulnerability to posttraumatic stress into account when developing routines for addressing the needs of intensified follow–up or preventive interventions for parents of high–risk pediatric patients.
